# The association between age at menarche and subsequent risk of chronic pain

**DOI:** 10.3389/fgwh.2026.1808092

**Published:** 2026-06-23

**Authors:** Sachiko Matsuzaki

**Affiliations:** 1 CH. U. Clermont-Ferrand, Chirurgie Gynécologique, Clermont-Ferrand, France; 2 Université Clermont Auvergne, Institut Pascal, UMR6602, CNRS/UCA/SIGMA, Clermont-Ferrand, France

**Keywords:** age at menarche, chronic pain, depression, gender difference, non-genetic factors

## Abstract

Chronic pain is a significant clinical issue worldwide. There is a well-documented gender difference in chronic pain, women experiencing it more frequently. Previous studies have shown that an earlier age at menarche is associated with an increased risk of chronic pain. Age at menarche can be altered by non-genetic factors, such as disturbances in the psychological or physical environment. Many studies have investigated non-genetic factors associated with the onset of menarche. However, no study has examined whether these factors, in conjunction with age at menarche, lead to chronic pain later in life. This review explores potentially shared non-genetic factors between age at menarche and subsequent chronic pain risk. There are potential links between age at menarche, non-genetic factors, and subsequent chronic pain risk. Studies have revealed that these links may be mediated by depression. Since information on age at menarche is easily obtainable, pediatricians, educators, school counselors, and psychologists can use it to identify girls at risk for chronic pain later in life. This review will raise awareness among these professionals, as well as other healthcare professionals, of the clinical importance of age at menarche in women with chronic pain. Pain perception varies among ethnic groups and is heavily influenced by culture. However, the majority of previously published studies were derived from “WEIRD” (Western, Educated, Industrialized, Rich, Democratic) populations. More research is needed to understand the relationship between age at menarche and subsequent chronic pain risk among women of different ethnicities.

## 1 Introduction

Chronic pain, which is defined as pain that persists for at least 3 months, is a significant clinical issue affecting over 30% of individuals worldwide (1, 2). It is a major public health concern because of the suffering it causes during daily activities, frequent absences from work, a substantial decline in quality of life, and high healthcare costs (1, 2). There is a well-documented sex difference in chronic pain, with a higher prevalence among women and girls (3–5). They often report experiencing more severe pain and greater functional impairment (3–5).

Previous studies have shown that an earlier age at menarche is associated with an increased risk of chronic pain (6–12). These studies have also suggested that girls who experience menarche at an earlier age have higher estrogen levels, which may increase the risk of chronic pain (6–12). However, both earlier menarche and delayed age at menarche might be linked to subsequent risk of chronic pain (13, 14).

In recent years, age at menarche has become a topic of growing interest due to its association with various psychological and physical outcomes (15, 16). Age at menarche is largely under genetic control. Genetic factors account for an estimated 57%–82% of the variation in age at menarche (17–19). However, this can be altered by non-genetic factors, such as disturbances in the psychological or physical environment (20). Numerous studies have investigated non-genetic factors associated with the onset of menarche (20). Several studies have revealed an association between age at menarche and an increased risk of chronic pain later in life (6–14). However, no study has examined whether these factors, in conjunction with age at menarche, lead to chronic pain later in life. This review explores the potentially shared non-genetic factors between age at menarche and the subsequent risk of chronic pain. Since age at menarche is easily accessible information, this information enables pediatricians, educators, school counselors, and psychologists to identify girls at increased risk for chronic pain and prevent these conditions. Additionally, a multidisciplinary approach to chronic pain management is necessary. However, not all healthcare professionals are familiar with the clinical importance of age at menarche in female physiology and disorders. This review may raise awareness among these professionals' of the clinical importance of age at menarche in girls and women with chronic pain. This information is also crucial for the personalized clinical management of women with chronic pain. Furthermore, this information suggests directions for future research investigating the biological mechanisms by which non-genomic factors influence the timing of menarche and lead to chronic pain later in life.

## 2 Literature search strategy and sources

A structured literature search strategy was designed to inform this narrative review and identify high-quality, relevant evidence associated with age at menarche and chronic pain in women. Peer-reviewed original and review articles written in English were selected from PubMed and Google Scholar searches up to January, 2026. The following keywords were used: Age at menarche; non-genomic factors; prenatal factors; parenting style; lifestyle factors; adverse childhood experiences; father absence; chronic pain, dysmenorrhea. The search was performed using these keywords individually and in combination. No restrictions were applied to the study design or publication date in order to ensure a comprehensive analysis. The inclusion criteria: Articles published within the past 10 years were prioritized, though significant older articles were also included. Retrospective and small-scale studies were included as well, but systematic reviews, meta-analyses, and large-scale prospective longitudinal studies were prioritized. The exclusion criteria: Studies involving animal subjects and *in vitro* experiments were excluded. Full-text articles were retrieved if their abstracts matched the inclusion criteria, or if it was unclear whether they did. The reference lists of the selected articles were manually examined to identify additional relevant publications. The articles were screened for eligibility based on clinical relevance. No formal quality assessments were conducted. Due to the limited number of studies investigating the relationship between age at menarche and chronic pain later in life, we first examined non-genetic factors that accelerate or delay age at menarche. Next, we examined the possibility of linking these factors to chronic pain later in life.

## 3 Results

### 3.1 Non-genetic factors that are associated with age at menarche

#### 3.1.1 Earlier age of menarche

Studies have revealed that several non-genetic factors are associated with an earlier age of menarche (21–64). These factors include:

- Prenatal factors, such as low birth weight, preterm birth, *in utero* exposure to diethylstilbestrol (DES) or tobacco, and prenatal exposure to maternal stress and emotional distress (21–32).
- Parenting style: maternal harsh control (35).
- Lifestyle factors: intake of sugar-sweetened and caffeinated beverages, an unhealthy diet, alcohol consumption between the ages of nine and ten, sedentary behavior, and shorter or longer sleep duration (36–43, 45–48).
- Adverse childhood experiences (ACEs) (50–56).
- Family structure: absence of a biological father, half- or step-siblings, and absence of biological parents and siblings (57–63).

#### 3.1.2 Later age at menarche

A later age at menarche is also associated with several non-genetic factors, including:

- Parenting style: maternal and paternal overprotection or neglect (33, 34).
- Lifestyle factors: physical activity or longer sleep duration (44, 49).
- ACEs (50, 52).
- Absence of a biological father (64).

### 3.2 Non-genetic factors associated with age at menarche and the risk of subsequent risk of chronic pain

Supplementary Table S1 summarizes studies that examined the relationship between non-genetic factors associated with age at menarche and the risk of subsequent risk of chronic pain.

#### 3.2.1 Prenatal factors

The Northern Finland Birth Cohort 1966 study revealed that preterm birth (before 37 weeks) rather than birth weight was associated with an increased risk of poor musculoskeletal health or low back pain in adulthood (65). A long-term follow-up study in Norway found that children in the very low birthweight (VLBW; <1,500 g) and small for gestational age (SGA) groups had higher odds ratios for chronic pain (66). However, an EU Horizon 2020 project revealed that the association between pain in adults is independent of birthweight or prematurity (67). Similarly, a retrospective cohort study in Germany found no link between prematurity and an increased risk of chronic pain in adults aged 15–52 (68). These results suggest an inconsistent relationship between experiencing chronic pain later in life and having a preterm birth or low birth weight. However, the study in Norway did not investigate the association between premature birth and chronic pain, nor did the study in Germany investigate the association between low birth weight and chronic pain. The participants in these four studies were all relatively young, ranging in age from 19 to 52 years old. Specifically, the Norwegian study (66) and the EU Horizon 2020 project (67) included patients between 19 and 28 years old. Chronic pain increases with age and becomes particularly common in the second half of life (69). Further studies of a large sample size including older populations are needed to investigate whether a preterm birth or a low birth weight is associated with the risk of developing of chronic pain in later life.

#### 3.2.2 Maternal and paternal parenting styles

Two studies of Japanese participants (70, 71) showed that the “affectionless control” parenting pattern (low care and high overprotection) was associated with chronic pain in adults. A study in the USA revealed that high paternal control and low maternal care lead to increased pain by exacerbating depressive symptoms in adolescents (72). A case-control study of Brazilian women aged 18–50 revealed that low maternal care was significantly more prevalent among women with chronic pelvic pain (CPP) than in the control group (60.7% vs. 45.2%, respectively). However, this association disappeared in the multiple regression analysis after adjusting for potential confounders (73). In this study, however, 24.8% of participants had no contact with their father for various reasons, which may have affected the results regarding paternal bonding and its potential link to CPP in women (73). These four studies suggested an association between low maternal care and chronic pain. Further studies are needed to validate these findings.

#### 3.2.3 Lifestyle factors

##### 3.2.3.1 Dietary patterns

There is a lack of research on the relationship between dietary patterns during childhood and adolescence and subsequent risk of chronic pain. However, several studies have revealed that adult dietary habits, such as consuming sugar-sweetened beverages (SSBs), are associated with an increased risk of chronic pain (74–77). Additionally, a two-sample MR analysis revealed that causal associations between various dietary habits and different types of chronic pain (78). Another two-sample MR analysis revealed a causal relationship between consuming artificially sweetened foods and experiencing chronic pain (79). Since studies have shown that eating behaviors remain stable over time, these dietary patterns may persist from childhood into adulthood and increase risk of chronic pain. Further studies are needed, however, to validate this assumption (80, 81). However, these findings are based on “WEIRD” (Western, Educated, Industrialized, Rich, Democratic) populations who consume a Western dietary pattern. Further research is needed to determine the impact of dietary patterns on age at menarche across a variety wide of ethnic and cultural groups.

##### 3.2.3.2 Physical activity/sedentary behavior

A systematic review revealed that physical activity during childhood and adolescence is linked with a reduced risk of multimorbidity later in life, including back pain (82). No longitudinal study has examined whether sedentary behavior during childhood and adolescence increases the risk of developing chronic pain later in life. However, a meta-analysis revealed that sedentary behavior is associated with a moderate increase in low back pain (LBP) risk in adults, children, and adolescents (83). Additionally, a meta-analysis of 20 Mendelian randomization studies revealed a causal effect of leisure sedentary behaviors on back pain (84). The effects of sedentary behavior on the risk of developing chronic pain may depend on the type of behavior and/or the timing of exposure (e.g., childhood vs. adulthood). Further longitudinal studies are needed to determine which types of sedentary behavior during childhood and adolescence increase the risk of chronic pain.

##### 3.2.3.3 Sleep disturbance

No study has examined the relationship between sleep duration or quality during childhood and the subsequent risk of chronic pain. However, there is some evidence supporting a bidirectional relationship between disrupted sleep and pain in children (85). Pain can inhibit sleep onset, and poor sleep can alter pain perception children (85). A meta-analysis of 20 Mendelian randomization studies revealed a causal effect of insomnia on back pain in adults (84).

#### 3.2.4 ACEs

A meta-analysis revealed that exposure to any direct ACEs, such as childhood sexual, physical, or emotional abuse or neglect, either alone or in combination with indirect ACEs, such as witnessing domestic violence or living with someone with a mental illness, significantly increases the odds of chronic painful conditions and pain-related disability in adulthood (86). The risk of chronic pain increases significantly from one ACE to four or more (86). A systematic review revealed that all types of ACEs are involved in the risk of chronic pain in adulthood (87). A recent cross-national study of from 22 countries representing various ethnicities and cultures found that individuals who experienced parental divorce, lived in a single-parent household, lost a parent, or suffered financial hardship or abuse during childhood were more likely to report pain later in life (88). A systematic review revealed an association between the number and severity of ACEs and an increased risk of dysmenorrhea (89). Sexual abuse and posttraumatic stress disorder were linked to dysmenorrhea, pelvic pain, and dyspareunia (89). Many studies have assessed conventional ACE domains using the 10-item ACE Study Questionnaire (90). However, because this questionnaire does not include bullying or financial hardships, no previous meta-analysis has evaluated the risk of chronic pain associated with these ACEs. However, a systematic review (91) and a cross-sectional study (92) revealed an increase in pain among those who were victimized by bullying (91). Studies from Finland have shown that experiencing financial hardship during childhood is associated with an increased risk of chronic pain conditions, including low back pain and fibromyalgia (93, 94). However, not all studies evaluated the influence of covariates. Studies have shown that poor mental health mediates the detrimental connection between ACEs and chronic pain (86, 89, 92). Therefore, mental health factors, such as depression and anxiety, should be considered important covariates.

#### 3.2.5 Father absence

Two cohort studies (93, 95) revealed an association between parental divorce or separation and a higher likelihood of experiencing chronic pain later in life. However, both studies lacked information on how many children continued to live with their mother, father, or other relatives (93, 95).

### 3.3 Age at menarche, non-genetic factors and depression

The present review suggests that poor mental health plays a role in the detrimental link between non-genetic factors associated with age at menarche and the subsequent risk of chronic pain later in life. Therefore, a literature search was conducted to determine if mental health is associated with age at menarche and if the aforementioned non-genetic factors associated with age at menarche and chronic pain are also associated with an increased risk of mental health issues.

#### 3.3.1 Causal associations between age at menarche and subsequent risk of poor mental health

Three meta-analyses revealed that girls who experienced early menarche were at an increased risk of developing depression later in life (96–98). Mendelian randomization studies have demonstrated a causal relationship between an earlier age at menarche and an increased risk of depression in adolescents and adults (99–103). However, since all participants in these MR analyses were of European ancestry, further studies are needed to investigate this relationship in a wide range of ethnic and cultural groups.

#### 3.3.2 Non-genetic factors associated with age at menarche and subsequent risk of depression

Previous studies have shown that several aforementioned non-genetic factors associated with age at menarche, including perinatal factors, parental styles, lifestyle factors, ACEs, and father absence, are also associated with an increased risk of depression (104–127).

#### 3.3.3 Causal links depression and chronic pain

Supplementary Table S2 summarizes the results of MR studies that examined the causal relationship between depression and the risk of developing chronic pain (85, 128–135). Several Mendelian randomization studies have revealed that depression is a cause of chronic pain (128–135). These studies have identified an association between depression and various types of pain, such as headaches, neck pain, shoulder pain, back pain, stomach pain, and abdominal pain (85, 128–131, 133, 134). They have also identified an association between depression and myalgia, fibromyalgia, osteoarthritis, and dysmenorrhea (132, 135). Some of these MR studies revealed that insomnia and short sleep duration are significant mediators of these associations (130, 132, 133).

## 4 Discussion

This review identified several non-genetic factors associated with age at menarche that are also associated with subsequent risk of chronic pain and depression. These factors include prenatal factors (preterm birth, very low birth weight, small for gestational age, and very preterm birth), parenting style (high paternal control), sedentary behavior, ACEs, and father absence. Furthermore, the review revealed that an earlier age at menarche is a causal factor for the subsequent risk of depression during childhood and/or adolescence. Depression mediates the link between ACEs and the subsequent risk of chronic pain (86, 89, 92). Women are twice as likely as men to experience depression (136, 137). These results support the hypothesis that an earlier age at menarche, in conjunction with the aforementioned non-genetic factors, can lead to depression during childhood and adolescence in some women. This depression can subsequently increase the risk of chronic pain in these women. Further studies are needed to investigate whether non-genetic factors causally affect the subsequent risk of chronic pain, as well as whether depression during childhood and/or adolescence mediates this link. This information could inform novel therapeutic strategies and targeted interventions that address mental health in children and adolescents.

Chronic pain disproportionately affects women (3–5). Sex differences in primary pain emerge during puberty, when females become more sensitive to painful stimuli and their rates of primary pain begin to rise dramatically Adolescent pain often persists into adulthood (138, 139). Therefore, recent studies have shown that interventions during adolescence are necessary to prevent chronic pain later in life (140). However, the biological mechanisms by which an earlier age at menarche leads to chronic pain later in life remain unclear.

Studies suggest that fluctuations in estradiol (E2) levels, rather than E2 levels, are major biological are major biological contributors to the development of gender differences in pain (141). Fluctuations in estrogen levels during adolescence are essential for pubertal development (142). Girls who experience menarche at an earlier age are exposed to these fluctuations sooner. This may explain why they are at a higher risk of developing chronic pain. However, studies have revealed that adaptive immune cells, particularly T lymphocytes, play a larger role in neuropathic pain in females (143, 144). These findings suggest that studies investigating the biological mechanisms by which an earlier age at menarche leads to chronic pain later in life should consider the various types of chronic pain. However, biological mechanisms are explained not only by fluctuations in estrogen levels and neuroimmune interactions, but also by psychosocial and socioeconomic factors (145). These factors may be complexly related to one another. Further research is necessary to understand how an earlier age at menarche increases the risk of chronic pain in later life (145).

Similarly, evidence from various fields indicates that sex hormones, particularly estradiol and progesterone, are significant contributors to gender differences in depression (146, 147). However, two MR analyses revealed no causal relationship between estrogen levels and depressive symptoms (148, 149). These two MR studies, however, did not investigate the effects of fluctuations of sex hormones on depressive symptoms. Research has indicated that estrogen fluctuations are a contributing factor to the increased risk of major depressive disorder (MDD) in women (150, 151).

A systematic review and meta-analysis of the effect of pubertal hormones on the risk of mental health problems in children and adolescents, including a total of 55 articles, criticized the fact that future research should measure not only absolute levels, but also the timing and tempo of hormonal changes (152). Such an analysis could better characterize how intra-individual hormonal changes impact mental health outcomes (152).

Chronic pain and depression are significant clinical issues that disproportionately affect women (3–5, 136, 145–147). Further studies investigating the biological mechanisms leading to chronic pain and depression later in life are needed to promote women's well-being. Understanding these mechanisms is crucial for developing strategies to prevent and treat chronic pain and depression. Additionally, further mechanistic studies are necessary to investigate potential connections between chronic pain and depression in individuals exposed to these non-genomic factors during childhood.

## 5 Limitations

This is the first narrative review to identify potential non-genomic, shared factors that influence both age at menarche and subsequent risk of chronic pain. However, I acknowledge the limitations of this review.

First, pain perception varies among ethnic groups and is heavily influenced by culture. However, the majority of the studies included in this review were derived from “WEIRD” populations. Therefore, it is unclear whether the current findings can be generalized to populations worldwide. Further studies that include a wide variety of ethnic and cultural groups are needed to confirm the findings. Second, adjusting for confounding factors is essential for making accurate causal inferences in observational studies. However, not all of the studies included in this review considered confounding factors. In particular, few studies considered mental health issues as confounding factors. Third, this review identifies lifestyle factors as one of the non-genetic factors associated with age at menarche. These modifiable factors are also associated with depression in children and adolescents. However, research on the relationship between dietary patterns, sleep duration, and sleep quality during childhood and adolescence, as well as their subsequent impact on chronic pain, is lacking. Further research is needed to investigate the association between modifiable factors during childhood and adolescence with subsequent chronic pain risk. This information will provide for the novel therapeutic strategies to prevent or reduce the subsequent risk of chronic pain through modify lifestyle factors during childhood.

## 6 Conclusion

This review identified potential non-genomic factors associated with age at menarche and the subsequent risk of chronic pain. However, not everyone exposed to these factors will have an earlier or later age at menarche. Therefore, it is crucial to effectively and carefully manage those exposed, regardless of their age at menarche.

Pain perception varies among ethnic groups and is heavily influenced by culture. However, the majority of previously published studies were derived from "WEIRD" populations. Further research is necessary to understand the relationship between age at menarche and subsequent chronic pain risk in women of different ethnicities.

## References

[1] Cohen SP Vase L Hooten WM. Chronic pain: an update on burden, best practices, and new advances. Lancet. (2021) 397:2082–97. 10.1016/S0140-6736(21)00393-7 34062143

[2] Kang Y Trewern L Jackman J McCartney D Soni A. Chronic pain: definitions and diagnosis. Br Med J. (2023) 381:e076036. 10.1136/bmj-2023-076036 37369384

[3] Osborne NR Davis KD. Sex and gender differences in pain. Int Rev Neurobiol. (2022) 164:277–307. 10.1016/bs.irn.2022.06.013 36038207

[4] Marchesini M Fornasari D Natoli S Vegni E Cuomo A. Sex-related differences in chronic pain: a narrative review by a multidisciplinary task force. Medicina. (2025) 61:1172. 10.3390/medicina61071172 40731802 PMC12298038

[5] eClinicalMedicine. Gendered pain: a call for recognition and health equity. EClinicalMedicine. (2024) 69:102558. 10.1016/j.eclinm.2024.102558 38486682 PMC10937548

[6] Wijnhoven HA De Vet HC Smit HA Picavet HSJ. Hormonal and reproductive factors are associated with chronic low back pain and chronic upper extremity pain in women–the MORGEN study. Spine. (2006) 31:1496–502. 10.1097/01.brs.0000220706.96724.76 16741461

[7] Aegidius K Zwart JA Hagen K Dyb G Holmen TL Stovner LJ. Increased headache prevalence in female adolescents and adult women with early menarche. The head-HUNT studies. Eur J Neurol. (2011) 18:321–8. 10.1111/j.1468-1331.2010.03143.x 20636369

[8] Bjelland EK Eberhard-Gran M Nielsen CS Eskild A. Age at menarche and pelvic girdle syndrome in pregnancy: a population study of 74 973 women. BJOG. (2011) 118:1646–52. 10.1111/j.1471-0528.2011.03099.x 21895953

[9] Kvalheim S Sandvik L Winsvold B Hagen K Zwart J-A. Early menarche and chronic widespread musculoskeletal complaints-results from the HUNT study. Pain. (2016) 20:458–64. 10.1002/ejp.747 26132558

[10] Kløven B Hoftun GB Romundstad PR Rygg M. Relationship between pubertal timing and chronic nonspecific pain in adolescent girls: the young-HUNT3 study (2006–2008). Pain. (2017) 158:1554–60. 10.1097/j.pain.0000000000000950 28520646

[11] Maleki N Kurth T Field AE. Age at menarche and risk of developing migraine or non-migraine headaches by young adulthood: a prospective cohort study. Cephalalgia. (2017) 37:1257–63. 10.1177/0333102416677999 27919016

[12] Lund CI Engdahl B Rosseland LA Stubhaug A Grimnes G Furberg A-S. The association between age at menarche and chronic pain outcomes in women: the Tromsø study, 2007 to 2016. Pain. (2022) 163:1790–9. 10.1097/j.pain.0000000000002579 35239542 PMC9393800

[13] Heuch I Heuch I Hagen K Storheim K Zwart J-A. Does the risk of chronic low back pain depend on age at menarche or menopause? A population-based cross-sectional and cohort study: the Trøndelag health study. BMJ Open. (2022) 12:e055118. 10.1136/bmjopen-2021-055118 35210341 PMC8883263

[14] Matsuzaki S Pouly JL Canis M. Analysis of risk factors associated with persistent noncyclic pelvic pain despite hormonal treatment in patients with endometriosis. Reprod Med Biol. (2025) 24:e12684. 10.1002/rmb2.12684 41221030 PMC12597974

[15] Lee JS Lee YA Shin CH Suh DI Yon DK. Long-term health outcomes of early menarche in women: an umbrella review. QJM. (2022) 115:837–47. 10.1093/qjmed/hcac187 35929081

[16] Lv Y Xia X Lei L Xiang W Wu X Xie S. Health outcomes of age at menarche in European women: a two-sample Mendelian randomization study. Postgrad Med J. (2023) 99:993–9. 10.1093/postmj/qgad023 37302123

[17] Anderson CA Duffy DL Martin NG Visscher PM. Estimation of variance components for age at menarche in twin families. Behav Genet. (2007) 37:668–77. 10.1007/s10519-007-9163-2 17680356

[18] Kaprio J Rimpelä A Winter T Viken RJ Rimpelä M Rose RJ. Common genetic influences on BMI and age at menarche. Hum Biol. (1995) 67:739–53. 8543288

[19] Morris DH Jones ME Schoemaker MJ Ashworth A Swerdlow AJ. Familial concordance for age at menarche: analyses from the breakthrough generations study. Paediatr Perinat Epidemiol. (2011) 25:306–11. 10.1111/j.1365-3016.2010.01183.x 21470270

[20] Yermachenko A Dvornyk V. Nongenetic determinants of age at menarche: a systematic review. Biomed Res Int. (2014) 2014:371583. 10.1155/2014/371583 25050345 PMC4094877

[21] Juul F Chang VW Brar P Parekh N. Birth weight, early life weight gain and age at menarche: a systematic review of longitudinal studies. Obes Rev. (2017) 18:1272–88. 10.1111/obr.12587 28872224

[22] Daniele C Wacks RE Farland LV Manson JE Qi L Shadyab AH. Associations between birthweight and preterm birth and the ages at menarche and menopause. BMC Womens Health. (2024) 24:546. 10.1186/s12905-024-03384-6 39363289 PMC11448270

[23] Itoi S Nagata C Piedvache A Morisaki N Ogawa K Yamamoto Y. Association between women’s birth weight and reproductive characteristics in adulthood: the JPHC-NEXT study. J Epidemiol. (2025) 35:432–41. 10.2188/jea.JE20240305 41047337 PMC12420951

[24] He R Liu R Wu H Yu J Jiang Z Huang H. The causal evidence of birth weight and female-related traits and diseases: a two-sample Mendelian randomization analysis. Front Genet. (2022) 13:850892. 10.3389/fgene.2022.850892 36035116 PMC9412024

[25] Wang L Xu F Zhang Q Chen J Zhou Q Sun C. Causal relationships between birth weight, childhood obesity and age at menarche: a two-sample Mendelian randomization analysis. Clin Endocrinol. (2023) 98:212–20. 10.1111/cen.14831 36237121

[26] Sabu S Corman H Noonan K Reichman NE Kuhn KB Radovick S. Small for gestational age and age at menarche in a contemporary population-based U.S. sample. PLoS One. (2024) 19:e0309363. 10.1371/journal.pone.0309363 39240976 PMC11379201

[27] Post A Veri S Moore D Poole M Kreider H Brunner Huber L. The association between early menarche and small for gestational age birth. J Biosoc Sci. (2023) 55:1039–43. 10.1017/S0021932023000020 36852575

[28] Suikkanen J Nurhonen M Cole TJ Paalanne M Matinolli H-M Tikanmäki M. Preterm birth and subsequent timing of pubertal growth, menarche, and voice break. Pediatr Res. (2022) 92:199–205. 10.1038/s41390-021-01690-5 34429512 PMC9411060

[29] Hatch EE Troisi R Wise LA Titus-Ernstoff L Hyer M Palmer JR. Preterm birth, fetal growth, and age at menarche among women exposed prenatally to diethylstilbestrol (DES). Reprod Toxicol. (2011) 31:151–7. 10.1016/j.reprotox.2010.11.006 21130156 PMC3057340

[30] Yermachenko A Dvornyk V. A meta-analysis provides evidence that prenatal smoking exposure decreases age at menarche. Reprod Toxicol. (2015) 58:222–8. 10.1016/j.reprotox.2015.10.019 26542102

[31] Houghton LC Goldberg M Wei Y Cirillo PM Cohn BA Michels KB. Why do studies show different associations between intrauterine exposure to maternal smoking and age at menarche? Ann Epidemiol. (2018) 28:197–203. 10.1016/j.annepidem.2018.01.004 29482744 PMC5975644

[32] Gaml-Sørensen A Brix N Henriksen TB Ramlau-Hansen CH. Maternal stress in pregnancy and pubertal timing in girls and boys: a cohort study. Fertil Steril. (2024) 122:715–26. 10.1016/j.fertnstert.2024.06.001 38848953

[33] Demakakos P Pashayan N Chrousos G Linara-Demakakou E Mishra GD. Childhood experiences of parenting and age at menarche, age at menopause and duration of reproductive lifespan: evidence from the English longitudinal study of ageing. Maturitas. (2019) 122:66–72. 10.1016/j.maturitas.2019.01.010 30797533 PMC6446039

[34] Li L Denholm R Power C. Child maltreatment and household dysfunction: associations with pubertal development in a British birth cohort. Int J Epidemiol. (2014) 43:1163–73. 10.1093/ije/dyu071 24706731 PMC4258777

[35] Belsky J Steinberg LD Houts RM Friedman SL DeHart G Cauffman E. Family rearing antecedents of pubertal timing. Child Dev. (2007) 78:1302–21. 10.1111/j.1467-8624.2007.01067.x 17650140

[36] Carwile JL Willett WC Spiegelman D Hertzmark E Rich-Edwards J Frazier AL. Sugar-sweetened beverage consumption and age at menarche in a prospective study of US girls. Hum Reprod. (2015) 30:675–83. 10.1093/humrep/deu349 25628346 PMC4325672

[37] Mueller NT Jacobs DR Jr MacLehose RF Demerath EW Kelly SP Dreyfus JG. Consumption of caffeinated and artificially sweetened soft drinks is associated with risk of early menarche. Am J Clin Nutr. (2015) 102:648–54. 10.3945/ajcn.114.100958 26178725 PMC4548172

[38] Davis CP Fest S Cushing-Haugen K Kensler TW Chavarro JE Harris HR. Dietary patterns and age at menarche in a prospective study of girls in the USA. Hum Reprod. (2025) 40:1087–93. 10.1093/humrep/deaf072 40328293 PMC12127513

[39] Duan R Chen Y Qiao T Zhao L Gong Y Cheng G. Modern dietary pattern is prospectively associated with earlier age at menarche: data from the CHNS 1997–2015. Nutr J. (2020) 19:95. 10.1186/s12937-020-00622-z 32907571 PMC7488069

[40] Nguyen NTK Fan HY Tsai MC Tung T-H Huynh Q Huang S-Y. Nutrient intake through childhood and early menarche onset in girls: systematic review and meta-analysis. Nutrients. (2020) 12:2544. 10.3390/nu12092544 32842616 PMC7551779

[41] Tang J Xue P Huang X Lin C Liu S. Diet and nutrients intakes during infancy and childhood in relation to early puberty: a systematic review and meta-analysis. Nutrients. (2022) 14:5004. 10.3390/nu14235004 36501034 PMC9739867

[42] Richards MA Oinonen KA. Age at menarche is associated with divergent alcohol use patterns in early adolescence and early adulthood. J Adolesc. (2011) 34:1065–76. 10.1016/j.adolescence.2010.11.001 21115194

[43] Ramraj B Subramanian VM. Study on age of menarche between generations and the factors associated with it. Clin Epidemiol Glob Health. (2021) 11:100758. 10.1016/j.cegh.2021.100758

[44] Calthorpe L Brage S Ong KK. Systematic review and meta-analysis of the association between childhood physical activity and age at menarche. Acta Paediatr. (2019) 108:1008–15. 10.1111/apa.14711 30588652 PMC6563453

[45] Puttawong D Wejaphikul K Thonusin C Dejkhamron P Chattipakorn N Chattipakorn SC. Potential role of sleep disturbance in the development of early puberty: past clinical evidence for future management. Pediatr Neurol. (2024) 161:117–24. 10.1016/j.pediatrneurol.2024.09.010 39368247

[46] Zhu MQ Mora-Plazas M Marín C Mora-Plazas M Villamor E. Sleep duration in middle childhood and age at menarche. Am J Hum Biol. (2023) 35:e23912. 10.1002/ajhb.23912 37171069

[47] Diao H Wang H Yang L Li T. The association between sleep duration, bedtimes, and early pubertal timing among Chinese adolescents: a cross-sectional study. Environ Health Prev Med. (2020) 25:21. 10.1186/s12199-020-00861-w 32560630 PMC7305621

[48] Ku SY Kang JW Kim H Jee BC Suh CS Choi YM. Age at menarche and its influencing factors in north Korean female refugees. Hum Reprod. (2006) 21:833–6. 10.1093/humrep/dei271 16199433

[49] Wang J Kwok MK Au Yeung SL Zhao J Lam HS Leung GM. Age of puberty and sleep duration: observational and Mendelian randomization study. Sci Rep. (2020) 10:3202. 10.1038/s41598-020-59811-9 32081851 PMC7035269

[50] Boynton-Jarrett R Harville EW. A prospective study of childhood social hardships and age at menarche. Ann Epidemiol. (2012) 22:731–7. 10.1016/j.annepidem.2012.08.005 22959664 PMC3469794

[51] Magnus MC Anderson EL Howe LD Joinson CJ Penton-Voak IS Fraser A. Childhood psychosocial adversity and female reproductive timing: a cohort study of the ALSPAC mothers. J Epidemiol Community Health. (2018) 72:34–40. 10.1136/jech-2017-209488 29122994 PMC5753025

[52] Boynton-Jarrett R Wright RJ Putnam FW Lividoti Hibert E Michels KB Forman MR. Childhood abuse and age at menarche. J Adolesc Health. (2013) 52:241–7. 10.1016/j.jadohealth.2012.06.006 23332491 PMC3950206

[53] Zhang L Zhang D Sun Y. Adverse childhood experiences and early pubertal timing among girls: a meta-analysis. Int J Environ Res Public Health. (2019) 16:2887. 10.3390/ijerph16162887 31412531 PMC6720214

[54] Ding W Xu Y Kondracki AJ Sun Y. Childhood adversity and accelerated reproductive events: a systematic review and meta-analysis. Am J Obstet Gynecol. (2024) 230:315–29. 10.1016/j.ajog.2023.10.005 37820985

[55] Su Q Chen Z Li R Elgar FJ Liu Z Lian Q. Association between early menarche and school bullying. J Adolesc Health. (2018) 63:213–8. 10.1016/j.jadohealth.2018.02.008 29705494

[56] Kubo A Aghaee S Acker J Deardorff J. Adverse childhood experiences are associated with the timing of puberty in girls but not in boys. J Adolesc Health. (2025) 76:1113–6. 10.1016/j.jadohealth.2025.02.009 40178462 PMC12162238

[57] Ellis BJ. Timing of pubertal maturation in girls: an integrated life history approach. Psychol Bull. (2004) 130:920–58. 10.1037/0033-2909.130.6.920 15535743

[58] Webster GD Graber JA Gesselman AN Crosier BS Schember TO. A life history theory of father absence and menarche: a meta-analysis. Evol Psychol. (2014) 12:273–94. 10.1177/147470491401200202 25299880 PMC10426907

[59] Culpin I Heron J Araya R Melotti R Lewis G Joinson C. Father absence and timing of menarche in adolescent girls from a UK cohort: the mediating role of maternal depression and major financial problems. J Adolesc. (2014) 37:291–301. 10.1016/j.adolescence.2014.02.003 24636689

[60] Smith D. O brother, where art thou? Investment in siblings for inclusive fitness benefits, not father absence, predicts earlier age at menarche. Biol Lett. (2017) 13:20170464; Erratum in: Biol Lett 2017; 13:20170650. 10.1098/rsbl.2017.0464 29046373 PMC5665773

[61] Lenárt P Zlámal F Kukla L Jarkovský J Bienertová-Vašků J. Sibling relatedness rather than father absence predicts earlier age at menarche in ELSPAC cohort. Biol Lett. (2019) 15:20190091. 10.1098/rsbl.2019.0091 31164060 PMC6597510

[62] Steppan M Whitehead R McEachran J Currie C. Family composition and age at menarche: findings from the international health behaviour in school-aged children study. Reprod Health. (2019) 16:176. 10.1186/s12978-019-0822-6 31805955 PMC6896716

[63] Guo S Lu HJ Zhu N Chang L. Meta-analysis of direct and indirect effects of father absence on menarcheal timing. Front Psychol. (2020) 11:1641. 10.3389/fpsyg.2020.01641 32849005 PMC7399376

[64] Sear R Sheppard P Coall DA. Cross-cultural evidence does not support universal acceleration of puberty in father-absent households. Philos Trans R Soc Lond B Biol Sci. (2019) 374:20180124. 10.1098/rstb.2018.0124 30966893 PMC6460089

[65] Heikkala E Chang JR Nieminen SS Vehkaperä K Kajantie E Karppinen J. Does birth weight or preterm birth predict worse pain prognosis in adulthood? A Northern Finland Birth Cohort study followed up to 46 years of age. J Pain. (2025) 27:104773. 10.1016/j.jpain.2024.104773 39743004

[66] Iversen JM Indredavik MS Evensen KA Romundstad PR Rygg M. Self-reported chronic pain in young adults with a low birth weight. Clin J Pain. (2017) 33:348–55. 10.1097/AJP.0000000000000399 27518485 PMC5348108

[67] Hollund IMH Aakvik KAD Benum SD Ingvaldsen SH Lydersen S Tikanmäki M. Mental health, pain and tiredness in adults born very preterm or with very low birthweight. Acta Paediatr. (2024) 113:72–80. 10.1111/apa.16982 37787099

[68] Tesarz J Schuster AK Hermes M Mildenberger E Urschitz MS Treede RD. Associations of preterm birth and neonatal stress exposure with chronic pain in adulthood—results from the gutenberg prematurity study. J Psychosom Res. (2024) 187:111943. 10.1016/j.jpsychores.2024.111943 39341156

[69] Calvo E Córdova C Shura R Allel K Alvaro C-C Keyes KM. Global pain and aging: a cross-sectional study on age differences in the intensity of chronic pain among middle-aged and older adults in 20 countries. J Gerontol B Psychol Sci Soc Sci. (2023) 78:1098–108. 10.1093/geronb/gbac199 36562345

[70] Anno K Shibata M Ninomiya T Iwaki R Kawata H Sawamoto R. Paternal and maternal bonding styles in childhood are associated with the prevalence of chronic pain in a general adult population: the Hisayama study. BMC Psychiatry. (2015) 15:181. 10.1186/s12888-015-0574-y 26227149 PMC4520085

[71] Shibata M Ninomiya T Anno K Kawata H Iwaki R Sawamoto R. Parenting style during childhood is associated with the development of chronic pain and a patient’s need for psychosomatic treatment in adulthood: a case-control study. Medicine. (2020) 99:e21230. 10.1097/MD.0000000000021230 32702896 PMC7373500

[72] Evans S Moloney C Seidman LC Zeltzer LK Tsao JCI. Parental bonding in adolescents with and without chronic pain. J Pediatr Psychol. (2018) 43:276–84. 10.1093/jpepsy/jsx110 29048481 PMC6454511

[73] Siqueira-Campos VM Fernandes LJH de Deus JM Conde DM. Parenting styles, mental health, and catastrophizing in women with chronic pelvic pain: a case-control study. Int J Environ Res Public Health. (2022) 19:13347. 10.3390/ijerph192013347 36293927 PMC9602934

[74] Strath LJ Brooks MS Sorge RE Judd SE. Relationship between diet and relative risk of pain in a cross-sectional analysis of the REGARDS longitudinal study. Pain Manag. (2022) 12:168–79. 10.2217/pmt-2021-0048 34431328 PMC8772533

[75] Wang Y Tang Y Li Z Jiang C Jiang W Hu Z. Sugar-sweetened beverage intake and chronic low back pain. Front Nutr. (2024) 11:1418393. 10.3389/fnut.2024.1418393 39021606 PMC11252024

[76] Liao Z Zeng J Chen Y Liu Z Zhou Z. Exploring the association between dietary caffeine and chronic musculoskeletal pain: a cross-sectional analysis of NHANES. Front Nutr. (2025) 12:1570403. 10.3389/fnut.2025.1570403 40357035 PMC12066468

[77] Zhang L Yin J Li J Sun H Liu Y Yang J. Association between dietary caffeine intake and severe headache or migraine in US adults. Sci Rep. (2023) 13:10220. 10.1038/s41598-023-36325-8 37353507 PMC10290098

[78] Zhou R Zhang L Sun Y Yan J Jiang H. Causal associations between dietary habits and chronic pain: a two-sample Mendelian randomization study. Nutrients. (2023) 15:3709. 10.3390/nu15173709 37686741 PMC10490345

[79] Zhao H Guan D Dong X Yao Y Ma Z. Causal effects of artificially sweetened foods on chronic pain mediated by gut microbiota: a Mendelian randomization study. Food Sci Nutr. (2025) 13:e70503. 10.1002/fsn3.70503 40552334 PMC12183110

[80] Chong MF. Dietary trajectories through the life course: opportunities and challenges. Br J Nutr. (2022) 128:154–9. 10.1017/S0007114522001295 35475441

[81] da Costa MP Severo M Araújo J Vilela S. Longitudinal tracking of diet quality from childhood to adolescence: the interplay of individual and sociodemographic factors. Appetite. (2024) 196:107279. 10.1016/j.appet.2024.107279 38401601

[82] Souilla L Larsen AC Juhl CB Skou ST Bricca A. Childhood and adolescence physical activity and multimorbidity later in life: a systematic review. J Multimorb Comorb. (2024) 14:26335565241231403. 10.1177/26335565241231403 38333053 PMC10851728

[83] Baradaran Mahdavi S Riahi R Vahdatpour B Kelishadi R. Association between sedentary behavior and low back pain; a systematic review and meta-analysis. Health Promot Perspect. (2021) 11:393–410. 10.34172/hpp.2021.50 35079583 PMC8767074

[84] Guan J Liu T Gao G Yang K Liang H. Associations between lifestyle-related risk factors and back pain: a systematic review and meta-analysis of Mendelian randomization studies. BMC Musculoskelet Disord. (2024) 25:612. 10.1186/s12891-024-07727-0 39090551 PMC11293147

[85] Oh A Koehler A Yonker M Troester M. Sleep disorders and chronic pain syndromes in the pediatric population. Semin Pediatr Neurol. (2023) 48:101085. 10.1016/j.spen.2023.101085 38065632

[86] Bussières A Hancock MJ Elklit A Ferreira ML Ferreira PH Stone LS. Adverse childhood experience is associated with an increased risk of reporting chronic pain in adulthood: a stystematic review and meta-analysis. Eur J Psychotraumatol. (2023) 14:2284025. 10.1080/20008066.2023.2284025 38111090 PMC10993817

[87] Nicolson KP Mills SEE Senaratne DNS Colvin LA Smith BH. What is the association between childhood adversity and subsequent chronic pain in adulthood? A systematic review. BJA Open. (2023) 6:100139. 10.1016/j.bjao.2023.100139 37588177 PMC10430872

[88] Macchia L Okafor CN Breedlove T Shiba K Piper A Johnson B. A cross-national analysis of childhood predictors of physical pain. Commun Med. (2025) 5:337. 10.1038/s43856-025-00997-2 40775017 PMC12331892

[89] Moussaoui D Grover SR. The association between childhood adversity and risk of dysmenorrhea, pelvic pain, and dyspareunia in adolescents and young adults: a systematic review. J Pediatr Adolesc Gynecol. (2022) 35:567–74. 10.1016/j.jpag.2022.04.010 35569788

[90] Felitti VJ Anda RF Nordenberg D Williamson DF Spitz AM Edwards V. Relationship of childhood abuse and household dysfunction to many of the leading causes of death in adults. The adverse childhood experiences (ACE) study. Am J Prev Med. (1998) 14:245–58. 10.1016/S0749-3797(98)00017-8 9635069

[91] Marin TJ Hayden JA Lewinson R Mahood Q Pepler D Katz J. A systematic review of the prospective relationship between bullying victimization and pain. J Pain Res. (2021) 14:1875–85. 10.2147/JPR.S313470 34188534 PMC8236267

[92] Varinen A Kosunen E Mattila K Suominen S Sillanmäki L Sumanen M. The association between bullying victimization in childhood and fibromyalgia. Data from the nationwide Finnish health and social support (HeSSup) study based on a sample of 64,797 individuals. J Psychosom Res. (2019) 117:48–53. 10.1016/j.jpsychores.2018.12.003 30665596

[93] Salonsalmi A Pietiläinen O Lahelma E Rahkonen O Lallukka T. Contributions of childhood adversities to chronic pain among mid-life employees. Scand J Public Health. (2022) 50:333–9. 10.1177/1403494820981509 33461395 PMC9096588

[94] Varinen A Kosunen E Mattila K Koskela T Sumanen M. The relationship between childhood adversities and fibromyalgia in the general population. J Psychosom Res. (2017) 99:137–42. 10.1016/j.jpsychores.2017.06.011 28712419

[95] Rouch I Strippoli MF Dorey JM Laurent B Ranjbar S Marques-Vidal P-M. Early-life adversity predicting the incidence of multisite chronic pain in the general population. Eur Psychiatry. (2024) 67:e67. 10.1192/j.eurpsy.2024.1753 39375924 PMC11536192

[96] Jiang L Hao Y Wang Y Chen Q Xin G Li P. Is early menarche related to depression? A meta-analysis. J Affect Disord. (2025) 369:508–15. 10.1016/j.jad.2024.10.036 39393462

[97] Ghadirzadeh E Moosazadeh M Shakeriastani K Zarrinkamar M Gheibi M Elyasi F. The association between age at menarche and depression: a systematic review and meta-analysis with meta-regression. Clinics. (2025) 80:100695. 10.1016/j.clinsp.2025.100695 40412096 PMC12148817

[98] Zhang C Yang J Yang W Cao P. Age at menarche and depression: an updated systematic review and meta-analysis. Arch Womens Ment Health. (2025) 28:983–95. 10.1007/s00737-025-01582-1 40175773 PMC12436512

[99] Askelund AD Wootton RE Torvik FA Lawn RB Corfield EC Magnus MC. Assessing causal links between age at menarche and adolescent mental health: a Mendelian randomisation study. BMC Med. (2024) 22:155. 10.1186/s12916-024-03361-8 38609914 PMC11015655

[100] Hirtz R Hars C Naaresh R Laabs B-H Antel J Grasemann C. Causal effect of age at menarche on the risk for depression: results from a two-sample multivariable Mendelian randomization study. Front Genet. (2022) 13:918584. 10.3389/fgene.2022.918584 35903354 PMC9315288

[101] Sequeira ME Lewis SJ Bonilla C Smith GD Joinson C. Association of timing of menarche with depressive symptoms and depression in adolescence: Mendelian randomisation study. Br J Psychiatry. (2017) 210:39–46. 10.1192/bjp.bp.115.168617 27491534 PMC5209630

[102] Wang Z Lu J Weng W Fu J Zhang J. Women’s reproductive traits and major depressive disorder: a two-sample Mendelian randomization study. J Affect Disord. (2023) 326:139–46. 10.1016/j.jad.2023.01.063 36682697

[103] Yu Y Hou L Wu Y Liu X He Y Ge Y. Causal associations between female reproductive behaviors and psychiatric disorders: a lifecourse Mendelian randomization study. BMC Psychiatry. (2023) 23:799. 10.1186/s12888-023-05203-y 37915018 PMC10621101

[104] O'Reilly EJ Mirzaei F Forman MR Ascherio A. Diethylstilbestrol exposure in utero and depression in women. Am J Epidemiol. (2010) 171:876–82. 10.1093/aje/kwq023 20332145 PMC2877444

[105] Burnett AC Anderson PJ Cheong J Doyle LW Davey CG Wood SJ. Prevalence of psychiatric diagnoses in preterm and full-term children, adolescents and young adults: a meta-analysis. Psychol Med. (2011) 41:2463–74. 10.1017/S003329171100081X 21733218

[106] Wojcik W Lee W Colman I Hardy R Hotopf M. Foetal origins of depression? A systematic review and meta-analysis of low birth weight and later depression. Psychol Med. (2013) 43:1–12. 10.1017/S0033291712000682 22717127 PMC3521225

[107] Loret de Mola C de França GV Quevedo Lde A de Avila Quevedo L Horta BL. Low birth weight, preterm birth and small for gestational age association with adult depression: systematic review and meta-analysis. Br J Psychiatry. (2014) 205:340–7. 10.1192/bjp.bp.113.139014 25368358

[108] Duko B Ayano G Pereira G Betts K Alati R. Prenatal tobacco use and the risk of mood disorders in offspring: a systematic review and meta-analysis. Soc Psychiatry Psychiatr Epidemiol. (2020) 55:1549–62. 10.1007/s00127-020-01949-y 32895729

[109] Upadhyaya S Sourander A Luntamo T Matinolli H-M Chudal R Hinkka-Yli-Salomäki S. Preterm birth is associated with depression from childhood to early adulthood. J Am Acad Child Adolesc Psychiatry. (2021) 60:1127–36. 10.1016/j.jaac.2020.09.020 33068750

[110] Gorostiaga A Aliri J Balluerka N Lameirinhas J. Parenting styles and internalizing symptoms in adolescence: a systematic literature review. Int J Environ Res Public Health. (2019) 16:3192. 10.3390/ijerph16173192 31480548 PMC6747480

[111] Richards G Smith A. Caffeine consumption and self-assessed stress, anxiety, and depression in secondary school children. J Psychopharmacol. (2015) 29:1236–47. 10.1177/0269881115612404 26508718 PMC4668773

[112] Kong W Gu J. Cross-sectional associations of lifestyle behaviors with depressive symptoms in adolescents. Int J Ment Health Promot. (2023) 25:139–52. 10.32604/ijmhp.2022.022123

[113] Liu J Chen T Chen M Ma Y Ma T Gao D. Sugar-sweetened beverages and depressive and social anxiety symptoms among children and adolescents aged 7–17 years, stratified by body composition. Front Nutr. (2022) 9:888671. 10.3389/fnut.2022.888671 35677554 PMC9168881

[114] Ding L Wu Z Wu Q Wei R Li E. Prevalence and lifestyle determinants of depressive symptoms among Chinese children and adolescents. Sci Rep. (2024) 14:27313. 10.1038/s41598-024-78436-w 39516529 PMC11607356

[115] Zhang E Chen J Liu Y Li H Li Y Kuwahara K. Associations between joint lifestyle behaviors and depression among children and adolescents: a large cross-sectional study in China. J Affect Disord. (2024) 352:110–4. 10.1016/j.jad.2024.02.032 38360364

[116] Haapala EA Leppänen MH Kosola S Appelqvist-Schmidlechner K Kraav S-L Jussila JJ. Childhood lifestyle behaviors and mental health symptoms in adolescence. JAMA Netw Open. (2025) 8:e2460012. 10.1001/jamanetworkopen.2024.60012 39951263 PMC11829227

[117] Lee SJ Na Y Lee KW. Association between the consumption of sugar-sweetened beverages and high-caffeine drinks and self-reported mental health conditions among Korean adolescents. Nutrients. (2025) 17:2652. 10.3390/nu17162652 40871678 PMC12389327

[118] Liu Q Peng S Jiang W He Y Lu C Wang W. Associations of black and sugar-sweetened coffee consumption with depressive symptoms: a longitudinal study of Chinese adolescents. J Affect Disord. (2025) 369:338–44. 10.1016/j.jad.2024.10.008 39368777

[119] Hughes K Bellis MA Hardcastle KA Sethi D Butchart A Mikton C. The effect of multiple adverse childhood experiences on health: a systematic review and meta-analysis. Lancet Public Health. (2017) 2:e356–66. 10.1016/S2468-2667(17)30118-4 29253477

[120] Bøe T Balaj M Eikemo TA McNamara CL Solheim EF. Financial difficulties in childhood and adult depression in Europe. Eur J Public Health. (2017) 27(suppl_1):96–101. 10.1093/eurpub/ckw253 28355649

[121] Elmore AL Crouch E. The association of adverse childhood experiences with anxiety and depression for children and youth, 8 to 17 years of age. Acad Pediatr. (2020) 20:600–8. 10.1016/j.acap.2020.02.012 32092442 PMC7340577

[122] Tan M Mao P. Type and dose-response effect of adverse childhood experiences in predicting depression: a systematic review and meta-analysis. Child Abuse Negl. (2023) 139:106091. 10.1016/j.chiabu.2023.106091 36787671

[123] Ye Z Wu D He X Ma Q Peng J Mao G. Meta-analysis of the relationship between bullying and depressive symptoms in children and adolescents. BMC Psychiatry. (2023) 23:215. 10.1186/s12888-023-04681-4 36997959 PMC10061722

[124] Yu P Jiang Z Zheng C Zeng P Huang L Jin Y. Variety ACEs and risk of developing anxiety, depression, or anxiety-depression co-morbidity: the 2006–2022 UK biobank data. Front Psychiatry. (2023) 14:1233981. 10.3389/fpsyt.2023.1233981 38234367 PMC10793109

[125] Matthews TA Shao H Forster M Kim I. Associations of adverse childhood experiences with depression and anxiety among children in the United States: racial and ethnic disparities in mental health. J Affect Disord. (2024) 362:645–51. 10.1016/j.jad.2024.07.121 39029666

[126] Auersperg F Vlasak T Ponocny I Barth A. Long-term effects of parental divorce on mental health—a meta-analysis. J Psychiatr Re. (2019) 119:107–15. 10.1016/j.jpsychires.2019.09.011 31622869

[127] Culpin I Heuvelman H Rai D Pearson RM Joinson C Heron J. Father absence and trajectories of offspring mental health across adolescence and young adulthood: findings from a UK-birth cohort. J Affect Disord. (2022) 314:150–9. 10.1016/j.jad.2022.07.016 35842065 PMC10666570

[128] Tang B Meng W Hägg S Burgess S Jiang X. Reciprocal interaction between depression and pain: results from a comprehensive bidirectional Mendelian randomization study and functional annotation analysis. Pain. (2022) 163:e40–8. 10.1097/j.pain.0000000000002305 34924553 PMC8675051

[129] Williams FMK Elgaeva EE Freidin MB Zaytseva OO Aulchenko YS Tsepilov YA. Causal effects of psychosocial factors on chronic back pain: a bidirectional Mendelian randomisation study. Eur Spine J. (2022) 31:1906–15. 10.1007/s00586-022-07263-2 35662366 PMC9273132

[130] Yao C Zhang Y Lu P Xiao B Sun P Tao J. Exploring the bidirectional relationship between pain and mental disorders: a comprehensive Mendelian randomization study. J Headache Pain. (2023) 24:82. 10.1186/s10194-023-01612-2 37415130 PMC10326936

[131] Zhao SS Holmes MV Alam U. Disentangling the relationship between depression and chronic widespread pain: a Mendelian randomisation study. Semin Arthritis Rheum. (2023) 60:152188. 10.1016/j.semarthrit.2023.152188 36963129

[132] Liu S Wei Z Carr DF Moraros J. Deciphering the genetic interplay between depression and dysmenorrhea: a Mendelian randomization study. Brief Bioinform. (2024) 26:bbae589. 10.1093/bib/bbae589 39592111 PMC11596086

[133] Zhu Y Bi Y Zhu T. Mendelian randomization highlights sleep disturbances mediated the effect of depression on chronic pain. Brain Behav. (2024) 14:e3596. 10.1002/brb3.3596 38967065 PMC11224770

[134] Liang H Wu Q Yang S Zhang S Miao J Jin H. Causal relationship between psychosocial factors and neck pain: a two-sample Mendelian randomization study. J Pain Res. (2025) 18:2191–201. 10.2147/JPR.S508287 40303578 PMC12039842

[135] Wei X Zhou T Wei X Wang H. Causal association between depression and myalgia-related disorders: a bidirectional Mendelian randomization study. J Affect Disord. (2025) 386:119450. 10.1016/j.jad.2025.119450 40398611

[136] Kuehner C. Why is depression more common among women than among men? Lancet Psychiatry. (2017) 4:146–58. 10.1016/S2215-0366(16)30263-2 27856392

[137] Kifle ZD Tian J Aitken D Melton PE Cicuttini F Jones G. Associations between endogenous sex hormones and multisite chronic musculoskeletal pain. Br J Anaesth. (2025) 134:793–803. 10.1016/j.bja.2024.11.021 39706703 PMC11867097

[138] Hagy H Hidalgo-Lopez E Portengen C Holman A Schrepf A Clauw DJ. The emergence of sex differences in primary pain during adolescence: a conceptual developmentally-oriented biopsychosocial model and opportunities for further investigation. BMC Pediatr. (2025) 25:899. 10.1186/s12887-025-06283-3 41188763 PMC12584504

[139] Murray CB Groenewald CB de la Vega R Palermo TM. Long-term impact of adolescent chronic pain on young adult educational, vocational, and social outcomes. Pain. (2020) 161:439–45. 10.1097/j.pain.0000000000001732 31651579 PMC7001863

[140] Jahre H Guddal MH Grasaas E Smedbråten K Riiser K Pripp AH. The effectiveness of school-based interventions on pain and disability outcomes in adolescents with persistent pain. A systematic review. Eur J Pain. (2025) 29:e70101. 10.1002/ejp.70101 40814866

[141] Athnaiel O Cantillo S Paredes S Knezevic NN. The role of sex hormones in pain-related conditions. Int J Mol Sci. (2023) 24:1866. 10.3390/ijms24031866 36768188 PMC9915903

[142] Shirtcliff EA Dahl RE Pollak SD. Pubertal development: correspondence between hormonal and physical development. Child Dev. (2009) 80:327–37. 10.1111/j.1467-8624.2009.01263.x 19466995 PMC2727719

[143] Mapplebeck JCS Beggs S Salter MW. Sex differences in pain: a tale of two immune cells. Pain. (2016) 157(Suppl 1):S2–6. 10.1097/j.pain.0000000000000389 26785152

[144] Ho MFS Farkas O Faria AV Plemel JR Kerr BJ. A recent history of immune cell sex differences in the peripheral nervous system in persistent pain states. Brain Behav Immun. (2025) 128:766–75. 10.1016/j.bbi.2025.05.004 40345628

[145] Bartley EJ Fillingim RB. Sex differences in pain: a brief review of clinical and experimental findings. Br J Anaesth. (2013) 111:52–8. 10.1093/bja/aet127 23794645 PMC3690315

[146] Li S Zhang X Cai Y Zheng L Pang H Lou L. Sex difference in incidence of major depressive disorder: an analysis from the global burden of disease study 2019. Ann Gen Psychiatry. (2023) 22:53. 10.1186/s12991-023-00486-7 38087353 PMC10714584

[147] Hankin BL Young JF Abela JR Smolen A Jenness JL Gulley LD. Depression from childhood into late adolescence: influence of gender, development, genetic susceptibility, and peer stress. J Abnorm Psychol. (2015) 124:803–16. 10.1037/abn0000089 26595469 PMC4662048

[148] Au Yeung SL Jiang C Cheng KK Zhang W Lam TH Leung GM. Genetically predicted 17beta-estradiol, cognitive function and depressive symptoms in women: a Mendelian randomization in the Guangzhou biobank cohort study. Prev Med. (2016) 88:80–5. 10.1016/j.ypmed.2016.03.002 27036929

[149] Oppenheimer H van der Meer D Schindler LS Crestol A Shadrin A Andreassen OA. No causal links between estradiol and female’s brain and mental health using Mendelian randomization. Nat Commun. (2025) 16:10915. 10.1038/s41467-025-65878-7 41350255 PMC12680690

[150] Albert KM Newhouse PA. Estrogen, stress, and depression: cognitive and biological interactions. Annu Rev Clin Psychol. (2019) 15:399–423. 10.1146/annurev-clinpsy-050718-095557 30786242 PMC9673602

[151] Kundakovic M Rocks D. Sex hormone fluctuation and increased female risk for depression and anxiety disorders: from clinical evidence to molecular mechanisms. Front Neuroendocrinol. (2022) 66:101010. 10.1016/j.yfrne.2022.101010 35716803 PMC9715398

[152] Luo D Dashti SG Sawyer SM Vijayakumar N. Pubertal hormones and mental health problems in children and adolescents: a systematic review of population-based studies. EClinicalMedicine. (2024) 76:102828. 10.1016/j.eclinm.2024.102828 39403116 PMC11472636

