# Supplementary material for: The association between age at menarche and subsequent risk of chronic pain
Source: Front Glob Womens Health. 2026 Jun 23;7:1808092. doi: 10.3389/fgwh.2026.1808092 (PMC13337901; doi:10.3389/fgwh.2026.1808092)
Supplement: Supplementary file 1 [file Table1.docx]

**Table 1: Summary of studies on non-genetic factors associated with age at menarche and the risk of subsequent chronic pain.**

| **Non genetic factors** | **Age at menarche** | **Reference** | **Country** | **Method** | **Age (y)** | **No of participants** | **Summary findings** | **Limitations** |
| --- | --- | --- | --- | --- | --- | --- | --- | --- |
| **Prenatal factors** |  |  |  |  |  |  |  |  |
| Preterm birth (<37 weeks) | Early | 65 | Finland | Longitudinal birth cohort | Followed from birth (born in 1966) until age 46. | 4871 participants (58% female) | Preterm birth appeared to predict assignment to the group with poorer musculoskeletal pain outcomes at age 46. However, birth weight did not predict these outcomes. | Neonatal care in the 1960s (before surfactant era) may have differed significantly from current practices, which could impact the results and their generalizability.  Self-reported pain.  No controls without pain |
| Very low birthweight (VLBW; <1500 g)  Term small gestational age (SGA) | Early | 66 | Norway | Longtudinal birth cohort | The mean age: 26, range 25-28 (born in 1986 -1988). | 216 (62 VLBW (30 female), 67 term SGA (30 female), and 87 controls (50 female) | The VLBW and SGA groups were at a higher risk of experiencing moderate to severe pain.  Adjusting for possible mediation by anxiety and depressive problems. | Small sample size.  The results may still be affected by unmeasured confounders. |
| Very preterm (VP; <32 weeks) and VLBW | Early | 67 | EU | 6 birth cohort studies |  | 617 born VP or VLBW (50.9% female) and 1122 term-born controls (55.8% female) | Mental health issues are linked to pain and fatigue in adults, regardless of whether they were born VP, VLBW or at term. | Three Finnish and one UK/Ireland cohort included only.  Muscle skeletal pain during the past six months, while the other two cohorts included any pain in the past four months.  The evaluation was conducted exclusively on young adults. |
|  |  |  | Finland |  | 26 (born in 1985-1986) |  |  |  |
|  |  |  | Germany |  | 26 (born in 1995) |  |  |  |
|  |  |  | UK an Ireland |  | 19 (born in 1978-85) |  |  |  |
|  |  |  | Finland |  | 23 (born in 1986-88) |  |  |  |
|  |  |  | Finland |  | 22 (born in 1985-1989) |  |  |  |
|  |  |  | Norway |  | 26 (born in 1986-1988) |  |  |  |
| Preterm birth (<37 weeks) | Early | 68 | Germany | Birth cohort | 18-52 (born in 1969-2002) | 439 participants (246 female) | Prematurity itself does not increase the risk of experiencing chronic pain later in middle adulthood. | Premature baby care differs between 1969-2002 (before surfactant era) |
|  |  |  |  |  |  |  |  |  |
| **Parenting style** |  |  |  |  |  |  |  |  |
|  | Late | 70 | Japan | Cross sectional | 39 to 92 (62% female) : | 760 (474 female): The prevalence of chronic pain was 46.4 % (48.3 % for women) | Paternal low care and high overprotection are associated with chronic pain in adults. | Self-reported, retrospective measure. |
|  | Late | 71 | Japan | Case control | Mean : Aproxilmately 50 yo | 150 with chronic pain 150 without pain  76% female | Parental low care and high overprotection are associated with an increased risk of chronic pain in the future. | No potential mediator analyzed.  No pain duration was assessed |
|  | Late | 73 | Brasil | Case control | 37.0 ± 6.9 (mean ± SD), controls: 31.9 ± 7.2 | 123 women with chronic pelvic pain and 123 pain-free controls. | Low maternal care was significantly more prevalent among women with chronic pelvic pain (CPP) than in the control group (60.7% versus 45.2%, respectively). However, this association disappeared in the multiple regression analysis after adjusting for potential confounders. | 24.8% of participants had no contact with their father for various reasons |
| **Life style factors** |  |  |  |  |  |  |  |  |
| Southern diet including sugar-sweetened beverages (SSB) | Early | 74 | USA | Cross sectional | 45-98 (mean 64.8) | 16,061 participants (55.4% female) | Southern’ dietary pattern was associated with a 41% (95% CI: 23, 61%) increase in RR of pain. | A bivariate pain variable was created using relevant yes/no questions from the self-reported data.  The present study also only assessed Black and non-Hispanic white participants |
|  |  |  |  |  |  |  |  |  |
| SSB | Early | 75 | USA | Cross sectional | 20-69 (mean 43.4) | 4,146 participants (50% female): with chronic low back pain: 14.4% | SSB consumption was significantly associated with an increased risk of chronic low back pain among individuals aged 20 to 69 years. | Data was obtained from dietary recall interviews and self-report questionnaires.  The sample size of participants with chronic low back pain was relatively small. |
| Caffeine | Early | 76 | USA | Cross sectional | Mean (SD) 50.1 (17.6) | 3,797 participants (51.4 female).  Chronic musculoskeletal pain prevalence of 18.41%. | Positive relationship between dietary caffeine intake and chronic musculoskeletal pain | Dietary caffeine intake was collected through a 24-h recall.  This study did not investigate the effects of specific caffeine-containing products (e.g., coffee, tea, chocolate) on chronic musculoskeletal pain. |
|  |  | 77 | USA | Cross sectional | Age ≥ 20 | 8993 adults (52.9% female) | Dietary caffeine intake is positively associated with severe headaches or migraines in US adults. | The data was obtained from questionnaires.  Severe headaches or migraines are based on self-reports.  The results may have been influenced by uncontrolled confounding factors.  The data is nearly 20 years old and may not reflect the current population. |
|  |  |  |  |  |  |  |  |  |
| Inflammatory diets | Early | 78 |  | Two sample MR | 38-73 y | Exposures:  20 different dietary habits; approximately 500,000 participants.  Outcomes: Multisite chronic pain (MCP):  387,649 European individuels.  MCP is defined as self-reported pain lasting at least three months in seven distinct bodily regions (head, face, neck/shoulder, back, stomach/abdomen, hip, and knee). | Causal associations between various dietary habits and different types of chronic pain.  Adhering to an anti-inflammatory diet, including increased consumption of fruit and cereal while reducing salt and pork intake, may potentially alleviate chronic pain symptoms. | The genome-wide association studies (GWAS) that were analyzed primarily focused on individuals of European ancestry, which may limit their applicability to other populations. |
| Artificially Sweetened Foods | Early | 79 |  | Two-sample MR |  | Artificially Sweetened Foods: UK Biobank GWAS: n=64, 949  Pain: atypical facial pain, thoracic pain, limb pain, joint pain, lowr back pain, sciatica: 8th version of the FinnGen consortium:  Cases: range: 1,508 (atypical facial pain)-189,683 (limb pain)  Controls: range: 221, 680-365,474  Genome-wide genotypes and 16S fecal microbiome data: the MiBioGen consortium:  18,340 individuals | There is a causal relationship between consuming artificially sweetened foods and experiencing chronic pain.  Consuming chocolate and flavored milk is associated with an increased risk of head and neck pain.  Drinks containing sugar are positively correlated with an increased risk of joint pain.  Eating sweet chocolate specifically increases the risk of sciatica.  Two-step Mendelian randomization analysis suggests that four types of gut microbiota have a mediating effect on three types of chronic pain: head and neck pain, joint pain, and sciatica. | There may be other potential confounding factors.  The genome-wide association studies (GWAS) that were analyzed primarily focused on individuals of European ancestry, which may limit their applicability to other populations. |
| Physical activity | Late | 82 |  | Systematic review | 7-55 | 11 studies (9 cross sectional, one retrospective longitudinal, one prospective longitudinal) | Reduced risk of multimorbidity later in life, including back pain | Low quality of evidence  Lack of repeated physical activity measurements throughout childhood and adulthood. |
| Sedentary behavior | Early | 83 |  | Meta-analysis | Mean: 9-65 | 27 studies:  sample size from 70 to 122377 | Sedentary behavior during work or leisure time is associated with a moderate increase in the risk of low back pain (LBP) in adults, children, and adolescents. | The definitions of LBP and the scales used to measure it varied considerably across the studies.  The definition and types of sedentary behavior vary across studies. |
| Leisure sedentary behaviors  Insommia | Early | 84 |  | Meta-analysis of MR studies investigating the association between various lifestyle factors and low back pain. |  |  | Positive causal effect of BMI, insomnia, smoking, alcohol consumption and leisure sedentary behaviors on back pain. | A patient-level analysis cannot be conducted due to the nature of a pooled analysis of individual MR studies.  There is potential for publication bias.  All of the studies are derived from data on individuals of European ancestry. |
| **ACEs** |  |  |  |  |  |  |  |  |
|  | Early or late  Most studies indicate that adverse childhood experiences (ACEs) are associated with an earlier age at menarche. However, a few studies have revealed that psychological and sexual abuse, verbal abuse, physical punishment, humiliation, witnessing abuse, family conflict, and maternal alcohol abuse are linked to an increased risk of delayed menarche. Additionally, cumulative childhood hardships have been associated with delayed menarche. | 86 |  | Meta-analysis | Mean: 44  range: 18–90 | 85 studies  826,452 adult participants | Individuals who were exposed to direct ACEs were significantly more likely to report chronic pain.  Individuals who reported experiencing physical abuse during childhood were significantly more likely to report chronic pain.  Exposure to any ACEs, either alone or in combination with indirect ACEs, significantly increases the odds of developing chronic painful conditions and experiencing pain-related disability in adulthood.  The risk of chronic pain increased significantly with each additional ACE. | The terminology and operational definitions of "child abuse and neglect," as well as the tools used to measure ACEs, vary across studies.  The accuracy of self-reported ACEs is uncertain.  Not all studies evaluated the influence of covariates.  The majority (84%) of included studies used a cross-sectional design. |
|  |  | 87 |  | Systematic review | 18≥ | 68 studies  196,130 participants | There are associations between ACEs and chronic pain in adulthood, and this relationship is dose dependent.  All types of ACEs should be considered risk factors for chronic pain in adulthood.  Poor mental health was found to mediate the detrimental connection between adverse childhood experiences and chronic pain. | Heterogeneity of terminology.  The majority of studies were retrospective.  Not all studies evaluated the influence of covariates. |
|  |  | 88 |  | Cross national  from 22 countries | 30–39 (20%), 40–49 (17%), 50–59 (16%) | 202,898 participants (49% female) | Individuals who experienced parental divorce, lived in a single-parent household, lost a parent, or suffered financial hardship or abuse during childhood were more likely to report pain later in life | The intensity, type, or interference of pain with daily activities could not be examined.  Recall bias.  Remaining unmeasured confounding, |
|  |  | 89 |  | Systematic review | Adolescents and young wemen | 19 studies (17 cross-sectional 2 case-control)  25,838 participants | An association was found between the number and severity of adverse childhood experiences (ACEs) and the risk of dysmenorrhea. Sexual abuse and posttraumatic stress disorder were associated with dysmenorrhea, pelvic pain, and dyspareunia.  No association was found for immigration or bullying. Findings were inconsistent for female genital mutilation, parental separation, and parental death. | It was unclear whether this relationship was mediated by poorer mental health.  The included studies used different definitions of the outcome. |
|  |  | 91 |  | Systematic review | 10-14 at baseline  follow-up periods: 0.5-12 | 4 longitudinal studies  6,275 participants | An increase in pain among those who were victimized by bullying | Unexplained heterogeneity  There is potential for reporting and publication biases due to the difficulty of publishing findings that show no connection between bullying victimization and pain. |
|  |  | 92 | Finland | Cross sectional | 20-54 | 11409 participans (61.9% female) having fibromyalgia (n = 515). | Statistically significant association between bullying victimization in childhood and fibromyalgia.  The association between bullying and fibromyalgia was not statistically significant when depression was included. | Self-reporting of fibromyalgia.  Recall bias.  Cross-sectional design with retrospective reports of childhood adversities. |
|  |  | 93 | Finland | Cross sectional | 40-60 | 8140 employees (80% women) | Chronic pain was associated with childhood economic difficulties, illness, parental divorce, parental alcohol problems, and bullying at school or among peers. | Cross-sectional design with retrospective reports of childhood adversities.  Under-response among younger employees, among those with lower occupational positions and among those with longer sickness absence |
|  |  | 94 | Finland | Cross sectional | 20-54 | 11409 participans (61.9% female).  having fibromyalgia (n = 515) | Chronic pain was associated with long-lasting financial difficulties; serious conflicts in the family; parental divorce; serious or chronic illnesses in the family; alcohol problems in the family | Self-reporting of fibromyalgia  Recall bias |
| **Family Structure** |  |  |  |  |  |  |  |  |
|  |  |  |  |  |  |  |  |  |
| Parental divorce or seperation | Early or late  Most of the studies revealed that the absence of a biological father is associated with an earlier age at menarche. However, these studies are based on WIRED (Western, Industrialized, Rich, Democratic) populations. Studies from non-WIRED populations, however, produced mixed results: early or late. | 93 | Finland | Cross sectional | 40, 45, 50, 55 or 60 | 8140 employees (79.9% female): Chronic pain was reported by 24% of men and 30% of women | Parental divorce or separation was associated with chronic pain in mid-life | Younger employees, among those with lower occupational positions and among those with longer sickness absence.  Lacked information on how many children continued to live with their mother, father, or others. |
|  |  | 95 | Switzerland | Cohort | 35-75 | 1537 participants (50.3% female) | A strong association was highlighted between parental divorce or separation and the risk of subsequent chronic pain (CP) with neuropathic characteristics | Because only cases of CP that occurred after the age of 40 were evaluated, we were unable to include subjects whose CP occurred during childhood, adolescence, or early adulthood.  Lacked information on how many children continued to live with their mother, father, or others. |
